# The ethnomedicinal evidences pertaining to traditional medicinal herbs used in the treatment of respiratory illnesses and disorders in Saudi Arabia: A review

**DOI:** 10.1016/j.sjbs.2022.103386

**Published:** 2022-07-22

**Authors:** Hamad Ghaleb Dailah

**Affiliations:** Research and Scientific Studies Unit, College of Nursing, Jazan University, Jazan 45142, Saudi Arabia

**Keywords:** Respiratory diseases, Traditional medicine, COVID-19, Herbs, Asthma, Bronchitis, Saudi Arabia

## Abstract

Due to their prevalence, respiratory diseases have attained great attention from the historical time. Furthermore, it has been explored in a new dimension due to recent viral outbreaks such as COVID-19. Even though modern medicine treats the majority of respiratory ailments, it is reported that the majority of people (≥80 %) who suffer from respiratory disorders do not take medication for their conditions, and a considerable number of people still believe in and use herbal medicines. Herbal therapies have been utilized all over the world for thousands of years. Traditional herbal treatment has long been seen as a valuable practice in Saudi Arabia, long before modern medicine. Due to its location in the desert and humid climate, Saudi Arabia suffers from a high rate of respiratory illnesses caused by dust, pollens, and viruses. Several published literature have employed different plants and plant products for respiratory problems, but there has yet to be a single, complete study centered on Saudi Arabia. In this review, 41 plants were identified, which has complete details regarding their usage in traditional practice for respiratory disorders. A thorough investigation was conducted and the results were detailed.

## 1 Introduction

In the domain of respiratory diseases, a wide array of conditions, such as asthma, chronic obstructive pulmonary disease (COPD), lung cancer, and several viral infections, may all be included under one comprehensive term. These disorders are usually accompanied by symptoms such as coughing, runny nose, sneezing, trouble breathing, bronchitis, influenza, common cold, sinus infections, and other similar conditions (Guo et al., 2021). In recent decades, respiratory infections have become a major public health concern in many parts of the world. According to official statistics, the number of people dying from respiratory disorders increased by 18 % between 1990 and 2020, despite the percentage of mortality attributed to respiratory diseases did not increase compared to other diseases over the same period. (Baptista et al., 2021). The Asian continent was the site of the vast majority of these deaths. As a result of the urbanization of major cities on the Asian continent in recent decades, tobacco smoking, industrial air pollution, allergies of various kinds, as well as bacterial and viral diseases such as COVID 19 have been identified as the primary causes of these respiratory diseases on the continent (Tsang and File Jr 2008). The vast majority of respiratory infections that occur in countries located in and around the Middle East are attributable to environmental causes. The hot warmth and dust of the desert, as well as the very low temperatures of the winter, make the sensitive populations more susceptible to respiratory disease than other groups (Waness et al., 2011). Respiratory disorders are common in Saudi Arabia. According to studies from the Ministry of Health in Saudi Arabia, respiratory disorders were regarded as the fifth most prevalent cause of mortality. Some of the significant elements that have been identified in Saudi Arabia as potential triggering factors for these disorders are certain pollens found in the desert region, cigar-smoking, dust, gases, biomass fuel, and air pollution, among others. This statistic has also been boosted by a number of virus outbreaks, such as MERS, which have occurred recently (Al Ghobain et al., 2011).

There has been a significant increase in the number of individuals taking herbal medications during the last few decades, despite the fact that herbal treatments have been used in many parts of the world for thousands of years. The market for herbal remedies, which may either be obtained via a doctor's prescription or taken care of by the patient themselves, is growing at a rapid rate. Even though many contemporary therapeutic agents are now accessible in clinical practice, many individuals still believe in and use herbal remedies. Traditional techniques have gained popularity as a result of the fact that they are both cost-efficient and have been shown to be successful over the course of time. (Younis et al., 2018). Traditional methods make use of a variety of different materials, the most prominent of which being plants; nevertheless, a great many others are also used. According to the World Health Organization, plant-based preparations are used to provide primary health care to more than 80 % of the world's population. Furthermore, these traditional plants or their synthetic analogs are responsible for the production of more than 10 % of the commercially available contemporary medications (Bogusz et al., 2002). The safety of traditional herbal plants is a key obstacle to their acceptance; most of them have not yet been well examined, and the vast majority of them do not have literature accessible on them. Because of this, it is important to create a database of such plants for future scientific investigations in order to explain their application and promote the creation of contemporary pharmaceuticals (Parveen et al., 2015).

**Fig. 1:**
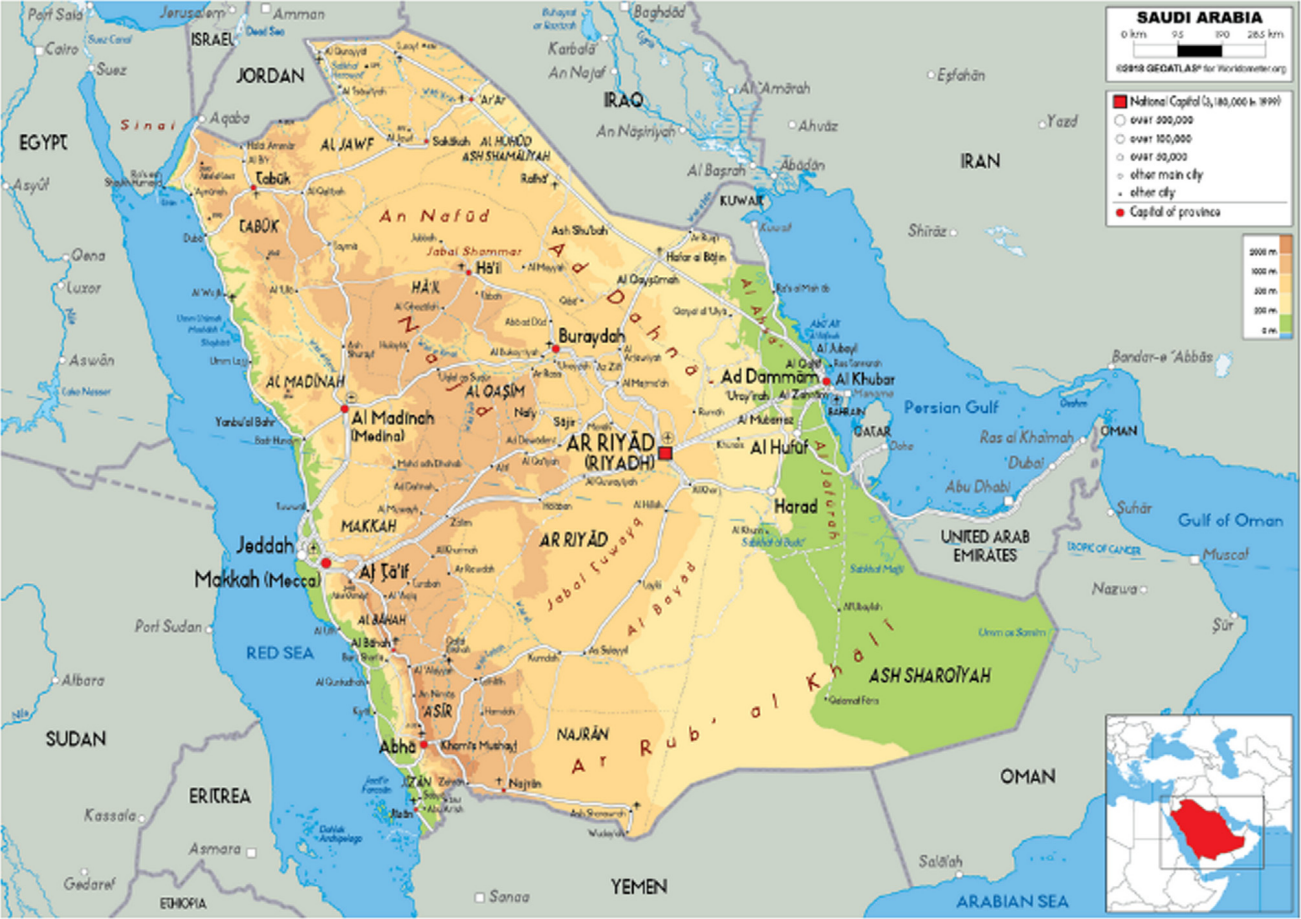
A map of Saudi Arabia's geography (map-saudi-arabia 2022).

The Kingdom of Saudi Arabia is located in the far southwest corner of Asia, and it shares borders with many nations in the Middle East, as well as Yemen and the Red Sea (Fig. 1). More than 2200 different types of plants may be found there. Since ancient times, Saudi Arabia has been known for herbs in traditional medicine as a significant practice. This was many centuries before the advent of modern medicine. The use of prophetic medicine in the treatment considerably reinforces its applicability. In the Middle East, Saudi Arabia is regarded as the origin of herbal medicine. Traditional healers in Saudi Arabia are referred to as Hakims, and they possess a plethora of indigenous knowledge on tribal and cultural medicine. They have herb collections that are geographically scattered around the nation. Saudi Arabia's terrain is varied, including mountains, forests, Greenland, and deserts. They all have various species, and some are only found in certain medicinally significant regions. Officially, these geographical locations are divided into provinces such as Al-Bahah, Al-Jawf, Asir province, Eastern province, Hail, Jizan, Madinah, Makkah, Najran, Qassim, Riyadh, and Tabuk. All of these provinces have their own particular plant species and customs. The majority of citizens of Saudi Arabia uses herbal drugs, which was very evident during the time of COVID-19 pandemic. AlNajrany et al., (2021) have found that during the COVID-19 pandemic, the Saudi population made extensive use of herbal supplements. Despite the fact that numerous plants have been used in traditional medicine to treat respiratory ailments, there is no complete report on the subject and only a handful have been studied in scientific investigations. As a result, the present study's goal is to collect data on the use of herbs for various respiratory illnesses in different Saudi Arabian areas using different sources.

## 2 Methodology

The data used in the analysis were obtained from a wide range of resources and databases, some of which include Google Scholar, PubMed, and Scopus. This investigation made use of a number of different keywords, some of which are as follows: herbs, plants, asthma, COPD, bronchitis, flu, cold, respiratory disorders, Saudi Arabia, traditional treatments, and local plants. The information was collected from a variety of sources, including but not limited to manuscripts, theses, books, book chapters, conference proceedings, and WHO reports published between the years 1956 and 2021. In the course of our research, we concentrated on 41 plants that (Table 1) were closely connected to the subject that was being discussed. Plant names that were incomplete or otherwise insufficiently documented were removed from the database. The taxonomic classification of the plants was validated with the help of the website https://www.worldfloraonline.org.

**Table 1:** List of traditional medicinal plants used to treat respiratory diseases.

**Sl no**	**Plants**	**Local name**	**Family**	**Parts used**	**Traditional use**	**Reference**
**1**	*Acacia arabica*(Lam.) Willd	Sant al arabi	Fabaceae	Whole herb	Used in treating cold and other respiratory diseases.	**(**Qari et al., 2021**)**
**2**	*Acalypha indica* L.	Thafilan	Euphorbiaceae	Whole herb	Used to cure bronchitis, pneumonia and asthma.	**(**Yusuf and Wahab 1994**)**
**3**	*Aloe vera* (L.) Burm. f.	Sabbar	Asphodelaceae	Whole herb	Used to cure asthma	**(**Qadir 2009**)**
**4**	*Alternanthera paronychioides* A.St.-Hil.		Amaranthaceae	Leaves	Used to cure bronchitis and asthma	**(**Saqib and Janbaz 2016**)**
**5**	*Anastatica hierochuntica* L.	Kaff	Brassicaceae	Whole plant	For the treatment of different respiratory conditions.	**(**Alqethami et al., 2020**)**
**6**	*Anethum graveolens* L.	Shabt	Apiaceae	Leaves & seed	Oral drink for respiratory diseases	**(**Alqethami et al., 2020**)**
**7**	*Artemisia judaica* L.	Beithran	Asteraceae	Leaves	Decoction for internal use to cure cough and cold	**(**Abdel-Kader et al., 2018**)**
**8**	*Aucklandia costus* Falc.	Qasd	Asteraceae	Root	Used in throat infections and respiratory infections as juice	**(**Alqethami et al., 2020**)**
**9**	*Azadirachta indica* A.Juss.	Neem	Meliaceae	Leaves	Leaves power mixed with honey for respiratory care	**(**Alqethami et al., 2020**)**
**10**	*Blepharis edulis* (Forssk.) Pers.	Al‑saha	Acanthaceae	Flower	Used for upper respiratory tract infection	**(**Ali et al., 2017**)**
**11**	*Blepharis ciliaris* (L.) B. L. Burtt	Al-Zaghaf	Acanthaceae	Leaves and roots	To treat cough and asthma, a decoction made of leaves, roots, and seeds is administered orally.	**(**Phondani et al., 2016, Tounekti et al., 2019**)**
**12**	*Cadaba farinose* (Forssk.)	Asaf	Capparaceae	Leaves	Coughs and other respiratory problems are treated using the juice of the leaves.	**(**Mossa et al., 1987**)**
**13**	*Capparis deciduas* (Forssk.) Edgew.	Tandhab	Capparaceae	Bark	The extract from the bark is used to treat coughs and asthma.	**(**Mossa et al., 1987**)**
**14**	*Cissus rotundifolia* Vahl	Gelef	Vitaceae	Leaves	Used to cure influenza	**(**Alshehri 2020**)**
**15**	*Commicarpus plumbagineus* (Cav.) Standl.	Roqma	Nyctaginaceae	Leaves	Used to treat asthma	**(**Qari et al., 2021**)**
**16**	*Datura metel* L.	Binj	Solanaceae	Leaves, roots and seeds	Used in treating asthma Dry leaves smoked for asthma and sinus infections	**(**Abulafatih, 1987, Rahman et al., 2004**)**
**17**	*Dipterygium glaucum* Decne.	Alqa	Capparidaceae	Whole herb	The plant is used in asthma management.	**(**Al-Shanwani 1996**)**
**18**	*Euphorbia hirta* L.;	Abana	Euphorpiaceae	Whole herb	A decoction made from the whole herb is used to treat allergic bronchitis and asthma.	**(**Yusuf and Wahab, 1994, Rahman et al., 2004**)**
**19**	*Euphorbia retusa* Forssk.	Na’maniah	Euphorbiaceae	Whole herb	Coughs and asthma are both treated with the whole herb.	**(**Al-Shanwani 1996**)**
**20**	*Euphorbia schimperiana* Scheele	Saibarisodis	Euphorbiaceae	Whole herb	A decoction made from the whole herb is used to treat allergic bronchitis and asthma.	**(**Rahman et al., 2004**)**
**21**	*Hyoscyamus muticus* L.	As-sakran	Solanaceae	Whole herb	Used for treating asthma	**(**Mossa et al., 1987**)**
**22**	*Juniperus procera* Hochst. Ex	Alar’ar	Cupressaceae	Leaves	Smoke of the Leaves used for cold and pharyngitis	**(**Ali et al., 2017**)**
**23**	*Jatropha glauca* Griseb.	Aobab	Euphorbiaceae	Stems	Dried and powdered decoction used in asthma	**(**Abdel-Kader et al., 2018**)**
**24**	*Launaea intybacea* (Jacq.)	Dahana	Asteraceae	Whole herb	Used in dry cough	**(**Chevallier 2016**)**
**25**	*Lavandula stoechas* subsp. Stoechas	Lavender	Lamiaceae	Whole herb	Used as expectorant and In asthma	**(**Mossa et al., 1987**)**
**26**	*Myrtus communis* L.	Al‑A’s	Myrtaceae	Leaves and bark	Used in asthma pharyngitis and cough	**(**Ali et al., 2017**)**
**27**	*Malva parviflora* L.	Khabiz	Malvaceae	Leaves and Roots	Used for cough	**(**El-Ghazali et al., 2010**)**
**28**	*Marrubium vulgare* L.	Frasyoon	Lamiaceae	Whole herb	A decoction made from the whole herb is used to treat allergic bronchitis and cough	**(**Nadkarni, 1996, Elberry et al., 2010**)**
**29**	*Mentha longifolia* (L.) L.	Naana	Lamiaceae	Leaves	The leaf extract is recommended as a treatment for cough and breathing issues	**(**Mossa et al., 1987**)**
**30**	*Monolluma quadrangular* (Forssk.)	Gelf	Asclepiadaceae	Leaves	Used in influenza	**(**Abdel-Kader et al., 2018**)**
**31**	*Nigella sativa* L.	Habbatus sauda	Ranunculaceae	Whole plant	Used for asthma	**(**Koshak et al., 2017**)**
**32**	*Ocimum basilicum* L.	Rahan	Lamiaceae	Leaves	Coughs may be alleviated by using the juice of the leaves.	**(**Rahman et al., 2004**)**
**33**	*Ocimum tenuiflorum* L., syn.	Shajrat-az-zir	Lamiaceae	Leaves	Cough and bronchitis may be alleviated by using the extract of the leaves	**(**Chopra et al., 1956**)**
**34**	*Origanum majorana* L.	Bardakush	Lamiaceae	Whole herb	Whole herb extract is used in the treatment of asthma and mild cough	**(**Rahman et al., 2004**)**
**35**	*Rosa abyssinica* R. Br	Al‑obal	Rosaceae	Fruits	Used in pharyngitis, cough Infusion	**(**Ali et al., 2017**)**
**36**	*Rumex nervosus* Vahl	Al‑athrub	Polygonaceae	Leaves	Used for asthma	**(**Ali et al., 2017**)**
**37**	*Solanum surattense* Burm.f.	Bankum	Solanaceae	Whole herb	Used for asthma	**(**Al-Shanwani 1996**)**
**38**	*Solanum coagulans* Forssk	Qitab	Solanaceae	Roots, Seeds.	Used for cough	**(**Koh et al., 2009**)**
**39**	*Thymus vulgaris* L.	Za’atar	Lamiaceae	Leaves	In cases of whooping cough, bronchitis, and the common cold, a leaf decoction is often used.	**(**Leung 1980**)**
**40**	*Trianthema portulacastrum* L.	Laani	Aizoaceae	Leaves and roots	Used for asthma	**(**Qari et al., 2021**)**
**41**	*Trichodesma africanum* L.	Hamham	Boraginaceae	Whole herb	Used for cough	**(**El-Ghazali et al., 2010**)**

## 3 Plants used traditionally for respiratory diseases

### 3.1 *Acacia Arabica* (Lam.) Willd

*Acacia Arabica* is one of the genus acacia's 1350 species. It is classified within the Leguminosae family, which Linnaeus initially categorized and named in 1773. This plant is also known as Indian gum, babool, and mimosa. It is an evergreen tree with a short trunk often seen in black or brown shades. It is common in regions with very hot climates, deserts, and arid terrain, particularly in the Arabian Peninsula, India, Africa, and Sri Lanka (Amadou et al., 2020). The plant may grow in all parts of Saudi Arabia, particularly in wadi habitats (valleys) and rowdahs (gardens) on sandy and alluvial soils. Locally, it is referred to as ‘sant al arabi’ and is a vital component of traditional medicine. It is used to treat a variety of respiratory tract illnesses, including the common cold (Qari et al., 2021). Additionally, this plant has significant effects against bacterial, viral, fungal and malarial infections. In addition, they are an excellent agent for ulcer protection, diabetes mellitus and an anti-oxidant. This plant's gum is abundant in phenolics and tannins. The fruit has a high concentration of phenolic acids, including m-digallic acid and gallic acid (Roqaiya et al., 2015).

### 3.2 Acalypha *indica* L

*Acalypha indica* is an erect annual plant of the Acalypha genus, which grows to a maximum height of 1.2 m. This plant belongs to the Euphorbiaceae family, and it's mostly found in Asia and Africa's wastelands and roadside. It grows mainly in Africa, such as Ethiopia and Somalia, and Asia, such as the Arabian Peninsula (Suresh et al., 2021). In Saudi Arabia, it is referred as “thafilan”, and it is used in conventional practice to cure bronchitis., pneumonia, and asthma (Yusuf and Wahab 1994). This plant has a significant history of use in traditional medicines all across the world, including as an anthelmintic, laxative, anti-epilepsy, headache treatment, anti-ulcer for bug bites, and wound healing. Analgesic, anthelmintic, anti-arthritis, anti-cancer, anti-fungal, antibacterial, anti-diabetic, anti-oxidant, anti-inflammatory, and anti-viral properties have been shown in scientific investigations. Fats, vitamins, minerals, and a variety of secondary metabolites of therapeutic use abound in this plant. It contains phenolics such as geraniin and corilagin, according to studies. Ellagic acid, gallic acid, kaur-en-18-oic-acid, 16,17-dihyroxy-ent-kauran 19-oic-acid, and 4,4′,5,5′,6,6′ hexahydroxy diphenic acid are all prevalent anti-oxidant chemicals. The presence of a molecule called quebrachitol is thought to be responsible for this plant's anti-asthmatic properties (Zahidin et al., 2017).

### 3.3 *Aloe vera* (L.) Burm. F

*Aloe vera* is one of the 400 species in the Aloe genus. It belongs to the Asphodelaceae family. It is thought that it originated on the Arabian Peninsula, but it has since spread quite far and is now regarded as an invasive species over the globe. It gets more vigorous in difficult circumstances and on terrain similar to a desert. It's a 100-cm-tall stemless or extremely short-stemmed plant with no leaves. It's found all across Saudi Arabia, particularly in the Asir deserts (Ameen et al., 2021). It's known as ‘Sabar’ in the area. In the traditional medicinal practice of Saudi, it is used to treat asthma (Qadir 2009). The gel of this plant is used for a variety of cosmetic purposes in many traditional practices. It was also approved for the treatment of cough, diabetes, cancer, arthritis, immune system problems, and inflammatory disorders. According to studies, it possesses anti-cancer, wound healing, anti-ulcer, anti-inflammatory, anti-diabetes, and anti-oxidant properties (Rajeswari et al., 2012). Aloe contains a lot of aloein, including nataloins like picric and oxalic acids, as well as a-barbaloins. Its antibacterial properties are due to the presence of lupeol and salicylic acid. Because of the inclusion of lupeol, it was an excellent analgesic (Maan et al., 2018).

### 3.4 Alternanthera *paronychioides* A.St.-Hil

*Alternanthera paronychioides* is a perennial plant with a prostrate stems that belonging to the Amaranthaceae family. Its natural habitat is the Mediterranean area. Its natural habitats include Asia, Australia, and Africa. It is also said to be prevalent throughout the Arabian Peninsula, namely in the United Arab Emirates and some areas of Saudi Arabia (Shahid and Rao 2015). According to their traditional medicine, the leaves of this plant are used to treat bronchitis and asthma in Saudi Arabia (Saqib and Janbaz 2016). Other traditional practices have included the use of this plant as a carminative and treatment for dermatitis. This plant has a high concentration of flavonoids, which contributes to its anti-oxidant properties (Erlejman et al., 2004). The presence of flavonoids has been shown to inhibit the production of free radicals, particularly ROS. This plant is well-known for its ability to stimulate beta cells in the pancreas and to lower blood sugar levels, both of which are beneficial in diabetics. As a result, it is regarded to be protective against glucotoxicity (Wu et al., 2013, Sufian et al., 2017).

### 3.5 Anastatica *hierochuntica* L

*Anastatica hierochuntica* L. is a tiny, grey-green winter annual plant of the Brassicaceae family. They grow to a maximum of 15 cm tall and have a distinctive white flower. Linnaei was the first to describe this plant in 1753 (Eshel et al., 2017). It is mostly found in the Arabian Peninsula, including the United Arab Emirates, Iraq, Jordan, Kuwait, and Saudi Arabia. It is called as 'Kaff' in Saudi Arabia, then in traditional medical practice, the plant itself is utilized to treat a variety of respiratory conditions. (Ihsanullah, 2012, Alqethami et al., 2020). The majority of Arabs consume this plant's leaves in the form of a tea beverage. In addition, the plant has been used to treat depression, hypertension, fever, malaria, epilepsy, hyperglycemia, as a cardiac medication, and in the treatment of infertility. Anti-oxidant, antibacterial, anti-fungal, hypolipidaemic, anti-inflammatory, hepatic protective and immunomodulation characteristics have been discovered in this plant. Minerals such as manganese, magnesium, calcium, and iron are abundant in the plant. Flavonoids, lignans, flavones, phenolic acids, flavanols, and other secondary metabolites are found in them. It also includes anti-oxidant substances such as kaempferol and ferulic acid (Zin et al., 2017).

### 3.6 *Anethum* graveolens L

*Anethum graveolens* L. is an annual with unique seeds and a distinct scent. It is a member of the Apiaceae family and may be found across Europe and southern Asia. This plant reaches a height of 18 in. and may now be found in a variety of locations all over the globe (Shaza et al., 2019). In Saudi Arabia, people often refer to this plant as “Shabat.”, and its seeds and leaves were used to make a drink and to treat respiratory problems in traditional medicine (Alqethami et al., 2020). The oil from this plant is very famous in other traditional practices as carminative, indigestion, mouth wash, aphrodisiac, anthelmintic etc. Several investigations have demonstrated that this herb has anti-fungal properties, antibacterial, anti-spasmodic, anti-oxidant, anti-inflammatory, diuretic, anti-tumor, anti-hypertensive, anti-tumor, and anti-hypertensive effects. So far, limonene, terpinol, carvonecaryophyllene, myristicin, and dillapiole have all been discovered as medicinally significant volatile oils (Shaza et al., 2019).

### 3.7 Artemisia *judaica* L

*Artemisia judaica* is a little shrub that grows to a height of 0.4 m. It is most often observed in Jordan's and Saudi Arabia's valleys and deserts. It is a member of the Compositae family. The presence of terpinoids gives these species' plants a distinct odor and flavor (Al-Qudah et al., 2021). In Saudi Arabia, it is referred as ‘Beithran’, where it is an important element of the traditional medical system. The areal plant is used to make a decoction that is used to cure coughs and colds (Abdel-Kader et al., 2018). It is effective in treating hepatitis, malaria, cancer, skin problems, GIT troubles, viral infections, and as anti-inflammatory drugs in different parts of the globe. This plant contains anti-microbial, analgesic, anti-pyretic, anti-cancer, anti-oxidant, anthelmintic, anti-fungal, and larvacidal properties, according to studies. Terpenoids, flavonoids, phenolic acids, and alkaloids are abundant in them (El-Amier et al., 2019).

### 3.8 Aucklandia *costus* Falc

The *Artemisia judaica* plant is a small shrub that may reach a height of up to 2 m when fully grown. It is native to the Mediterranean region. This plant can be found largely in the Himalayas, and it is also growing in certain parts of Pakistan (Lee et al., 2018). It belongs to the Asteraceae family and is referred as 'Qasd' in Saudi Arabia. Local traditional medicine practitioners, particularly in Jeddah province, utilize the juice extracted from the roots of this plant to treat throat infections and respiratory infections (Alqethami et al., 2020). Other ancient medical techniques include the treatment of gastro-intestinal discomfort, as an anti-inflammatory, ear, nose, and throat infections, as well as asthma, epilepsy, typhoid fever, and scabies (Pandey et al., 2007). According to pharmacological investigations conducted on this plant, it has anti-inflammatory, anti-ulcer, liver protection, anti-tumor, immunomodulatory, anti-fungal, and antibacterial properties. The chemical profile of this plant indicates that it includes a variety of secondary metabolites. The presence of Cynaropicrin, costunolide, and other anti-cancer compounds is thought to be responsible for the anti-cancer action (Nadda et al., 2020).

### 3.9 Azadirachta *indica* A.Juss

*Azadirachta indica* A.Juss. is a tall tree that may reach 20 m in height. It is an evergreen tree that is native to the Indian subcontinent and may be found all the way up to Kenya. It is a member of the Meliaceae family and has a number of therapeutic properties (Islas et al., 2020). The Arafat region of Saudi Arabia is where the plant is cultivated, which has the world's biggest plantation of this tree (Ahmed et al., 1989). It's known as ‘Neem’ in the area, and the powdered leaves of this plant are traditionally blended with honey to treat coughs and other respiratory ailments (Alqethami et al., 2020). It is used as an anti-cancer, anti-hypertensive, anti-coronary disease, anti-diabetes, anti-fungal, and other traditional medicines. It possesses anti-oxidant, anti-inflammatory, anti-diabetic, and anti-cancer properties, according to studies. Nimbin, a triterpenes phytochemical, is responsible for the majority of the plant's pharmacological actions. Flavonoids in the plant have anti-inflammatory properties. Limonoids, tannins, alkaloids, terpenoids, and catechins are among the additional substances found in the plant (Islas et al., 2020, Braga et al., 2021).

### 3.10 Blepharis *edulis* (Forssk.) Pers

*Blepharis edulis* is a tiny perennial plant that grows in dry and semi-arid environments and has simple and broad leaves. This plant is a member of the Acanthaceae family and has a self-supporting growth type. Pakistan and Morocco are habitats to this species (Ashour 2015). On the other hand, it is easily accessible in a number of other nations like Kenya, India, and Saudi Arabia. In Saudi Arabia, they call it “Alsaha,” and an infusion made from the plant's flowers is used to cure respiratory infections. (Ali et al., 2017). This herb is also used to treat asthma, cough, fever, as a purgative, anti-inflammatory, stomach ulcer, wound healing agent, and as an aphrodisiac in various traditional practices. Studies show that the plant exhibits significant activity against microbes such as fungal infection, against cancer and inflammation. It has potent anti-oxidant and anti-dysentery properties, according to studies. It contains benzoxazine glucoside, banzoxazolone, and blepharin, according to phytochemical study. The presence of phenolic compounds and glycosides is responsible for the majority of the pharmacological actions (Saqib et al., 2012, Mahboubi et al., 2013).

### 3.11 Blepharis *ciliaris* (L.) B. L. Burtt

*Blepharis ciliaris* is an annual plant that is found in semi-arid regions and may reach a height of up to 60 cm in the semi-arid climate of the Mediterranean. It is a member of the Acanthaceae family, and it is native to Saudi Arabia, however, it is also found in Iran, Pakistan, and the Sultanate of Oman. In Saudi Arabia, where it is known as ‘Al-Zaghaf’, this plant is both significant and endemic, and it is a valuable resource. Oral administration of a decoction made of the plant's leaves, roots, and seeds is, according to traditional medicine practice, an effective treatment for cough and asthma. (Phondani et al., 2016, Tounekti et al., 2019). Aside from sores and inflammation, it is utilized in various traditional medical practices to alleviate cough, diuretic effects and to treat high fevers, wound healing and gastro-intestinal disorders. Investigations using pharmacological methodologies have shown, among other beneficial effects, that it inhibits the growth of microbes and cancer cells and reduces inflammation. Plant phytochemicals such as 7-O-(3-acetyl-6-ethyl-coumaroylglucoside), apigenin-7-O-glucoside, acteoside, and protocatechuic acid have been isolated and studied in more detail (El-Shanawany et al., 2013, Dirar et al., 2021).

### 3.12 Cadaba *farinosa* Forssk

*Cadaba farinosa* Forssk. is a slender, evergreen shrub with a deeply wrinkled stem. It is a member of the family Capparaceae and can be distributed to different locations across the globe, particularly in tropical areas (Al-Musayeib et al., 2013). Rocky regions along Saudi Arabia's coastline in the south west, Azan-al-arnab, and Al Baha are the primary locations for the plant's cultivation. People refer to it as ‘Asaf’ in the region, and it's utilized in traditional medicine to treat coughs and respiratory ailments (Mossa et al., 1987). It was also used for diarrhea, cough, anthelmintic, stimulant, rheumatic pain, amenorrhea, antiphlogistic, and worm infestations in traditional medicine. Research carried out in the area of pharmacology has shown that it has significant activity against inflammation and type-2 diabetes. It also has shown activities such as purgative, hepatoprotective, and anthelmintic. It contains a variety of secondary metabolites such as alkaloids, phenolics, fatty acids, and other compounds, including the medicinally important lupeol, stachydrine, and quercetin (Telrandhe et al., 2010).

### 3.13 Capparis *deciduas* (Forssk.) Edgew

*Capparis deciduas* (Forssk.) Edgew. (Family: Capparaceae) is a perennial bushy shrub or tree that grows up to 5 m in height and is mostly found in sub-tropical and tropical climates. It is cultivated in Saudi Arabia in a few isolated locations, including Raudhat Khuraim near Riyadh. ‘Tandhab’ is the local name for it, and traditional healers utilize its bark extract to cure asthma and cough (Mossa et al., 1987). Cough, toothache, dysentery, diarrhea, skin rashes, constipation, stomach ulcers, renal disorders, and rheumatism are some of the additional traditional uses. This plant contains anti-rheumatic, anti-oxidant, anti-fungal, anti-gout, insecticidal, anti-viral, anti-inflammatory, analgesic, antibacterial, and anti-platelet effects, according to pharmacological investigations. Several secondary metabolites, including alkaloids, flavonoids, glycosides, and pure chemicals like Spermidine, capparisinine, and cadabicine, have been isolated from this plant, with many therapeutic effects (Abdel-Mawgood et al., 2005, Nazar et al., 2020).

### 3.14 Cissus *rotundifolia* Vahl

*Cissus rotundifolia* Vahl is an endemic shrub to the Arabian Peninsula and Africa. The Vitaceae family includes this plant, which is widely grown in Saudi Arabia's southern region, particularly in the Asir province (AL-Bukhaiti et al., 2019). It's called in the region as 'Gelef,' and its leaves have long been used to cure influenza (Alshehri 2020). Furthermore, it is utilized to treat diabetes, dermatological disorders, and burns. This herb is also used for gastro-intestinal issues, liver illnesses, fever, and to promote appetite in other traditional therapies. Furthermore, this plant is said to be a good provider of nutrients. Pharmacological research has shown that it has the potent property of curing diabetes, cancer and osteoporosis properties. Triterpenoids, phenolics, flavonoids, stilbene derivatives, iridoids, and coumarin glycosides are among the secondary metabolites isolated from it (Said et al., 2015, Korish, 2016).

### 3.15 Commicarpus *plumbagineus* (Cav.) Standl

*Commicarpus plumbagineus* is a perennial plant that grows up to 10 m tall and produces annual stems from a woody base. It comes from Africa and belongs to the Nyctaginaceae family. Many parts of the globe, including Africa, Madagascar, the Arabian Peninsula, Palestine, Israel, Jordan, and India, have access to it. It is widely available in the Asir and Taif districts of Saudi Arabia, where it is regarded as 'Roqma.' This plant's leaves are used in indigenous Saudi Arabian medicine to treat asthma (Qari et al., 2021). It's also used as an emetic, to cure jaundice, as an expectorant, as a laxative, and to treat ulcers, burns, and swollen glands. This plant has been shown to have anti-microbial and immunomodulatory properties in studies. There haven't been many researches done to investigate its impacts and chemical profile. An examination of the plant's phytochemistry revealed the presence of secondary metabolites such as glycosides, saponins, terpinoids, sterols, and nitrogen-containing substances (Ojok, 2007, Ojok et al., 2007).

### 3.16 *Datura metel* L

*Datura metel* L., sometimes known as Thornapple, is a short-lived shrub that belongs to the family Solanaceae. It may be found in India and Africa in warm climates. It may also be found in Kenya, Tanzania, and Uganda. It is located across Saudi Arabia, notably in the desert area near Riyadh and in eastern regions (Taha and Mahdi 1984). It is known as ‘Binj’ in Saudi Arabia, and the leaves, roots, and seeds are used in traditional medicine to treat asthma. For asthma and sinus infections, dry leaves were smoked (Abulafatih 1987). Bronchitis, ulcers, psoriasis, inflammation, skin rashes, jaundice, and diabetes are other conditions that may be cured with the leaves in various traditional treatments. This plant contains anti-arthritis, insecticidal, herbicidal, anti-fungal, anti-cancer, antibacterial, anti-diabetic, and anti-oxidant activities, according to pharmacological investigations. The presence of tropane alkaloids in this plant is critical. It has a lot of alkaloids in it. Valtropine, fastusine, atropine, and scopulamines are among the chemicals that make this plant medicinally significant (Monira and Munan 2012).

### 3.17 Dipterygium *glaucum* Decne

*Dipterygium glaucum* Decne. is a perennial branching shrub that belongs to the Capparidaceae plant family. Egypt, Sudan, Pakistan, and Saudi Arabia are among the countries where this plant may be found in abundance. This plant is called as ‘Alqa’ in the local language, and it may be found in abundance in the shrubby and sandy area of the Tihama plains, which are on the route between Jeddah and Mecca. As a bronchodilator, this plant is used to treat asthma in the local traditional medical system (Al-Shanwani 1996). In other traditional traditions, it is used to treat psoriasis, ringworm, jaundice, and as a blood purifier, among other things. Medical research has shown that this plant has anti-oxidant, anti-microbial, and cancer-fighting capabilities. Flavonoids, phenolics, tannins, proanthocyanidins, Stigmasterol, -sitosterol, -sitosterol, Campesterol, and Squalene were found in the plant, according to the results of the phytochemical examination (Moussa et al., 2012).

### 3.18 Euphorbia *hirta* L

*Euphorbia hirta* L. is an annual hairy plant that may reach a height of 50 cm and belongs to the family Euphorpiaceae. This plant originated in the tropical areas of the Americas and has since expanded across the tropics and subtropics at low altitudes. It may be found in Saudi Arabia in a variety of locations, most notably in Riyadh and Yambu, where it is known as ‘Abana’. As a plant infusion, it plays a significant role in the practice of traditional Saudi medicine, and it is used to treat respiratory conditions, including bronchitis and asthma (Yusuf and Wahab, 1994, Rahman et al., 2004). This herb is also used for conjunctivitis, inflammatory disorders, dysentery, diarrhea, parasite infection, cancer, skin illness, snake bites, and boils in various traditional treatments. It possesses anti-inflammatory, anti-malarial, anti-cancer, anti-asthmatic, anti-diarrhea, anti-fertility, and anti-fungal properties, according to several pharmacological researches. Secondary metabolites such as Afzelin, euphorbin, quercitrin, sitosterol, and camphol were found in the phytochemical examination. Amino acids and other nutritious components abound in this plant (Kumar et al., 2010, Awaad et al., 2017).

### 3.19 Euphorbia *retusa* Forssk

*Euphorbia retusa* Forssk (Family: Euphorbiaceae) is a kind of perennial plant that may reach a height of 60 cm and has the look of being both glabrous and glaucous. This plant may be found growing in many places around the globe. Its natural habitats include Mauritania, North Africa, Jordan, and the Arabian Peninsula, among other places. It is known as ‘Na'maniah’ in Saudi Arabia and it is found in large numbers on the plains, particularly in the Qassim region of the country. According to traditional medical practices in the area, it is used to treat cough and asthma (Al-Shanwani 1996). Other traditional methods recommend it for the treatment of eczema, warts, and inflammatory diseases. According to pharmacological research, this plant contains excellent properties against cancer, inflammation, viruses, and diabetes mellitus, among other things. The presence of flavanol glycosides, triterpinoids, diterpinoids, polyphenols, flavonoids, and tannins, among other things, was discovered by phytochemical research (Sdayria et al., 2018, Lahmadi et al., 2020).

### 3.20 Euphorbia *schimperiana* Scheele

*Euphorbia schimperiana* Scheele is a kind of plant that may either be an annual or a perennial and has several branches. It belongs to the family Euphorbiaceae and can reach a height of up to 2 m. It is native to Europe and Asia. Its natural habitats include tropical Africa and the Arabian Peninsula. The plant is known as Saibarisodis in Saudi Arabia, and it is only found in small numbers in the Asir and western heights of the country. Coughs, asthma, and other respiratory problems are treated with a decoction of the herb, which is utilized in traditional medicine (Rahman et al., 2004). Others use it for cancer prevention, ear pain relief, anti-snake venom, and inflammatory diseases, among many other applications. Pharmacological investigations have shown that it has anti-viral, anti-oxidant, and cytotoxic properties. Studies on the phytochemical composition have shown the existence of diterpenoids, triterpenoids, and flavonoids, including the presence of 3-Cycloartenol, Chrysin, and Qurecetin, among others (Shaker et al., 2015, Aljubiri et al., 2021).

### 3.21 Hyoscyamus *muticus* L

*Hyoscyamus muticus* L. is a species of plant that may either be a biennial or a short-lived perennial. It can reach a height of 0.6 m and belongs to the family Solanaceae. It is native to the Mediterranean region. Syria, Afghanistan, Pakistan, and Saudi Arabia are the most common places to witness it. It is referred to as ‘As-sakran’ in the local language, and it may be found largely along the northern border and in the Tabuk area. As an asthma treatment, traditional healers in Saudi Arabia use the whole plant, including the roots and leaves (Mossa et al., 1987). The usage of this plant in various traditional practices included the treatment of Parkinson's disease, gastro-intestinal problems, motion sickness, and as a smooth muscle relaxant. There has been relatively little research done on this plant, and it is still under investigation. Pharmacological research revealed that it had anti-fungal and antibacterial properties. This plant has the potential to cause hallucinations and has some poisonous qualities; therefore, it should be used with caution. Chemical substances such as ferrulic acid, methyl salicylate, and methyl ferulate were discovered in phytochemical research (Elsharkawy et al., 2018).

### 3.22 *Juniperus* procera Hochst. Ex

*Juniperus procera* Hochst. Ex is a tree species that may reach a height of 40 m and belongs to the Cupressaceae family. Although it is endemic to Ethiopia and Eastern Africa, it may also be found in Saudi Arabia. It is particularly common in Saudi Arabia's desert area, where it is known as ‘Alar'ar.' The leaves of this plant are smoked and used in local traditional medicine to treat colds and pharyngitis (Ali et al., 2017). The leaves are also used to treat diabetes, constipation, rheumatism, headaches, and many skin ailments in other traditional treatments. This tree's resin is combined with honey and used to treat ulcers and preserve the liver. This plant's essential oil has been shown to be anti-oxidant. It has anti-fungal and anti-microbial properties. This tree contains alkaloids, flavonoids, sterols, diterpenes, tannins, and volatile oil, according to phytochemical studies (Burits et al., 2001, Salih et al., 2021).

### 3.23 *Jatropha glauca* Griseb

The *Jatropha glauca* is a blooming plant that may reach a height of 6 m and has numerous branches and blooms. It is a member of the Euphorbiaceae family, and it is indigenous to North Africa and the Arabian Peninsula. In diverse places of the globe, such as Eritrea, Somalia, Sudan, Ethiopia, Yemen and the Al-Baha region of Saudi Arabia, this plant may be spotted growing wild. Traditional healers in Saudi Arabia utilize a decoction produced from the dried stem of this plant for internal use to treat asthma patients (Abdel-Kader et al., 2018). In Saudi Arabia, they refer to it as “Aobab.”. This plant is used in a variety of therapeutic circumstances as an astringent to alleviate ear pain in patients and to treat constipation, among other purposes. Pharmacological research has shown that this plant is anti-oxidant and has significant activity against microbes, cancer and viruses among other activities. The phytochemicals steric acid, oleic acid, linoleic acid, and palmitic acid, which have been isolated from this plant, are among the most significant (Albasha et al., 2015, Aati et al., 2018).

### 3.24 *Launaea intybacea* (Jacq.)

*Launaea intybacea* (Jacq.) is an annual plant that may reach a height of up to 150 cm and is considered a weed. The area around the Mediterranean is where it was first discovered. This plant is a member of the Asteraceae family and is indigenous to Africa. Other continents, such as America and Asia, are replete with examples of this phenomenon. Known as ‘Dahana’ in Saudi Arabia, dry coughs may be alleviated with the help of this herb, which is used in traditional medicine and is common in the country (Chevallier 2016). It is also used in the treatment of liver illnesses, as a blood cleanser, in the treatment of skin diseases, in the treatment of dyspepsia, and as a galactogogue, among other things. A number of research investigations have shown that this plant contains hepatoprotective and anti-oxidant properties. The phytochemical investigation confirmed the existence of lupeol, oleanolic acid, coumaric acid, and apigenin, among other constituents of the plant (Takate et al., 2010, Malik, 2012, Saleem et al., 2013).

### 3.25 *Lavandula stoechas* subsp

*Lavandula stoechas* subsp. is a woody evergreen shrub that grows to a height of 6 feet and has flowers in summer. It belongs to the Lamiaceae family and is endemic to Europe. It is quite unusual to see in Saudi Arabia, however, it may be found in the Albaha area, where it is referred to as ‘lavender’. It has long been used to cure asthma and as an expectorant (Mossa et al., 1987). Other traditional traditions recommend it for the treatment of rheumatic disorders and renal problems. It has also been reported to be used as a pain reliever, in inflammatory conditions, and as anti-spasmodic. Pharmacological research revealed that it has antibacterial, anti-inflammatory, anti-oxidant, and anti-leishmanial properties, among other things. Flavonoids, alkaloids, sterols, terpinoids, glycosides, and tannins have been discovered in this plant, according to phytochemical investigations (Boufellous et al., 2017, Khayyat et al., 2018, Bousta and Farah, 2020).

### 3.26 *Myrtus communis* L

*Myrtus communis* L. is a species of evergreen, perennial shrub that may reach heights of up to 5 m and is a member of the family Myrtaceae. This plant is native to Europe, North Africa, and the Asian subcontinent. In Saudi Arabia, people refer to this plant as “Al-As,” and it may be found in the Al-Qassim region. Since ancient times, different parts of this plant have been used in Saudi Arabian cuisine and traditional medicine for the treatment of asthma, pharyngitis, and cough therapy using infusions of leaves and bark. These applications date back to the country's agricultural beginnings (Ali et al., 2017). It is also used for depression, hypertension, eczema, dandruff, sinus infection, sleeplessness, stomach discomfort, and anxiety in various traditional treatments. It possesses anti-microbial, anti-diabetes, anti-cancer, anti-ulcer, anti-diarrheal, anti-inflammatory, and sedative activities, according to pharmacological investigations. Phytochemical analyses demonstrated the presence of phenolics, flavonoids, terpinoids, quercitin, catechin, and gallocatechin, among other secondary metabolites (Sisay and Gashaw, 2017, Alyousef, 2021).

### 3.27 *Malva parviflora* L

*Malva parviflora* L. is an annual or perennial herb that is a member of the Malvaceae family of plants. Originally from North Africa, it is now found across Europe and Asia. It may be found in the Qassim Region of Saudi Arabia, where it is known as ‘khabiz’ in the native language. Traditional practitioners in Saudi Arabia use the leaves and roots of this plant to cure coughing and other respiratory ailments (El-Ghazali et al., 2010). In various traditional treatments, it is used to heal wounds, ulcers, inflammation, and bruised and broken limbs, as well as to prevent infection. Studies on the plant’s pharmacological effects have shown that it possesses significant activity in diabetes mellitus, inflammatory conditions, in bacterial and fungal infections. It also showed as an excellent anti-oxidant. It contains a variety of secondary metabolites, including phenols, flavonoids, esters, and peptides, all of which have been discovered. It is a hazardous plant, and a substance contained inside it known as Malvalic acid has been determined to be the cause of its toxicity (Shale et al., 2005, Al-Otibi et al., 2021).

### 3.28 Marrubium vulgare L

Originally from Europe, Africa, and Asia, *Marrubium vulgare* is a perennial aromatic shrub with a strong fragrance. It belongs to the family Lamiaceae and may reach a height of up to 50 cm at its tallest point. Known as ‘frasyoon’ in Saudi Arabia, it is readily available and popular among the general public, mostly seen in the Asir province. Traditionally, in Saudi Arabia, whole herb paste is used for the treatment of the common cold, TB, jaundice, as skin preparation, chronic bronchitis and cough, among other uses (Elberry et al., 2010). Other traditional practices recommend it for the treatment of jaundice, piles, and diarrhea. It has several advantages in terms of pharmaceutical benefits, including anti-oxidant, anti-inflammatory, and anti-hypertensive properties. It is also beneficial in the treatment of diabetes, asthmatic stomach disorders and bacterial infections. It contains a significant quantity of secondary metabolites, a few examples of which include phenolics, flavonoids, and essential oils, among many more. The compounds arenarioside, acteoside, and forsythoside B, which were isolated from this plant, were shown to have remarkable anti-cancer and anti-inflammatory activity. Marrubinic acid, which is found in the plant, has choleretic properties (Lodhi et al., 2017, Aćimović et al., 2020).

### 3.29 Mentha *longifolia* (L.) L

*Mentha longifolia* (L.) L. is a perennial plant that grows to a height of 1 m and has a wide range of uses. Its natural habitats include Europe, Africa, and Asia. It is a member of the Lamiaceae family and may be found in a variety of locations, including Afghanistan, Iraq, China, Iran, and Saudi Arabia. It is known as ‘naana’ in Saudi Arabia, and it may be found in the western mountainous parts of the country as well as the Albaha Area in southern Saudi Arabia. The leaf extract is used as a cough treatment in the local traditional medicine system (Mossa et al., 1987). In various ancient traditions, it is used to treat asthma, cough, cold, as sedative, kidney stone, headache, diuretic, constipation, and indigestion, among other conditions. The results of the pharmacological investigations revealed that it possesses nerve-protecting, anti-oxidant, and anti-cancer, anti-helmintic, immunomodulatory, antibacterial, anti-hemolytic, and muscle relaxant properties. Numerous medicinally essential compounds, including as pulegone, menthone, isomenthone, and menthol, have been discovered in the plant via phytochemical investigations (Mossa et al., 1987, Mikaili et al., 2013, Farzaei et al., 2017).

### 3.30 Monolluma *quadrangular* (Forssk.)

*Monolluma quadrangular* is a succulent shrub with irregularly branching stems and leaves that may grow up to 60 cm in height. It belongs to the Asclepiadaceae family, which is endemic to the Arabian Peninsula where it may be found naturally. Traditional healers roast the leaves over coals to soften them, then crush them to lower the amount of water they contain before cooking them with spices and serving them to patients to treat influenza (Abdel-Kader et al., 2018). It is locally known as ‘gelf’. In various practices, it is used as an anti-ulcer agent, to treat diabetes mellitus, skin rashes, snake bites, inflammation, cancer, and to prevent cancer. The results of pharmacological tests revealed that it had significant activity against diabetes, ulcer and inflammatory conditions. According to the results of a phytochemical investigation, it includes sterols, terpenoids, alkaloids, and flavonoids, among other things (Bin-Jumah, 2018, Bin-Jumah, 2019).

### 3.31 Nigella *sativa* L

*Nigella sativa* L. is an annual blooming plant native to Asia and the Mediterranean. It is a member of the Ranunculaceae family and may grow to a height of 30 cm in height. The seed of this plant is well-known for its therapeutic properties. In Saudi Arabia, it is referred to as ‘habbatus’ or ‘sauda’. When it comes to treating asthma, traditional medicine practitioners use the whole plant (Koshak et al., 2017). The treatment of hepatitis, diabetes, wound healing, osteoarthritis, inflammation and liver problems, as well as the prevention and treatment of malaria and infections, are also employed in traditional therapies. Pharmacological investigations have shown that it has anti-microbial, hepatoprotective, anti-diabetic, anti-inflammatory, anti-infertility, anti-cancer, anthelmintic, analgesic, anti-hypertensive, and anti-fungal characteristics, as well as anti-infection and anti-fungal qualities. The seeds of this plant included a variety of phytoconstituents, including terpene alcohol, alkaloids such as nigellimin and thymoquinone, as well as a number of different fatty acids (Sharma et al., 2009, Majeed et al., 2021).

### 3.32 Ocimum *basilicum* L

*Ocimum basilicum* L. is a fragrant annual or perennial herb that may grow to a height of 0.5 m. It has a strong basil flavour and can be used in cooking. It is a member of the Lamiaceae family and is indigenous to India. It may be found all across the globe, from Africa to the Southeast and even in Saudi Arabia, among other places. In Saudi Arabia, it may be found in the Al Madinah Al Munwara region, the southern Hijaz region, and the eastern province, where it is known as ‘rahan’. Cough is treated with the help of the leaves of this plant, according to local traditional medicine practices (Rahman et al., 2004). The herb is also used in various traditional practices to treat diabetes, cardiovascular issues, headaches, coughs, renal ailments, snake and bug bites and to relieve pain. It is also used to treat sedatives and painkillers. Pharmacological investigations have shown that this plant has significant activity in microbial infections, diabetes mellitus, in epilepsy, inflammatory conditions and in cancers. It also possesses immunomodulatory and antiplatelet activities. Some of the most important phytochemicals found in plants are camphor, limonene, thymol, citral, Methyl eugeno, anthocyanins, limonene, thymol, myrcene, and borneol (Purushothaman et al., 2018, Shahrajabian et al., 2020).

### 3.33 Ocimum *tenuiflorum* L., syn

*Ocimum tenuiflorum* L syn is an erect annual or short-lived aromatic perennial plant native to the tropical and subtropical Indian subcontinent. It is intensely perfumed and grows to a height of 0.6 m. It is a member of the Lamiaceae family. It is prevalent in a large number of Asian nations, including Saudi Arabia. It is mostly found in Saudi Arabia's Al Madinah Al Munwara province, where it is locally referred to as ‘shajrat-az-zir’. Traditionally, leaf extracts of this plant were used to treat coughs and bronchitis on a local level (Chopra et al., 1956). It is also used in various traditional practices to treat headaches, fevers, runny noses, colds, heart disorders, inflammation, malaria, and poisoning. Pharmacological research has shown that it has anti-diabetic, cardioprotective, anti-oxidant, anti-cancer, anti-microbial, anti-ulcer, immunomodulatory, anti-infertility, analgesic, and anthelmintic activities. Numerous medicinally significant active compounds have been identified in this plant, including Eugenol, urosolic acid, carvacrol, linalool, and estragol (Pattanayak et al., 2010).

### 3.34 Origanum *majorana* L

*Origanum majorana* L. plant is a perennial shrub that may reach a height of up to 60 cm in certain cases. It is a member of the Lamiaceae family, and its natural habitats are in both Europe and North America. In Saudi Arabia, it may be found in a variety of locations in the country's southern areas, including Al-Soda, where it is known as ‘bardakush’. It is used to treat asthma and cough in Saudi Arabia, when the whole plant is consumed (Rahman et al., 2004). In various traditional practices, it is used as an anti-allergic, in the treatment of flu and fever, as an anti-pyretic, as an anti-diabetic, in the treatment of menstruation pain, as an anti-spasmodic, and in the treatment of stomach discomfort. Several clinical and laboratory research have shown that it has anti-microbial, anti-parasitic and anti-oxidant characteristics. It is also an effective diabetes and cancer prevention agent as well as an anti-inflammatory and anti-mutagenic agent. This plant has been used to extract a variety of phytochemicals, including thymol, tannis, arbutin, sitosterol, apigenin, luteolin, and camphene, among others (Bouyahya et al., 2021).

### 3.35 Rosa *abyssinica* R. Br

*Rosa abyssinica* R. Br is an ever green shrub considered as a creeper or climber that grows to a height of 7 m. It is a member of the Rosaceae family and is indigenous to Africa. This plant may also be found in other parts of the world, including Ethiopia, Ethiopian Republic, Yemen, and Saudi Arabia. In Saudi Arabia, it is most often found in the Al-Soda and Asir provinces. It is referred to as ‘al-obal’ in the local language, and in traditional medicine, the infusion of the fruits of this plant is used to treat pharyngitis and cough (Ali et al., 2017). Other traditional practices include the treatment of high blood pressure, the prevention of worm infestations, the treatment of TB, the use of the herb as an anti-diabetic medicine, and the treatment of cough. Pharmacological investigations have shown that it possesses immunomodulatory, anti-hypertensive, anti-inflammatory, analgesic, and anti-microbial properties, among other properties. Polyphenolic chemicals, tannins, flavonoids, and steroid compounds were discovered in the plant's secondary metabolites, according to phytochemical studies. A significant number of medicinally important phytochemicals, including squalene, ß-D-glucopyranose, and furfural, have been discovered and identified (Sewuye and Asres, 2008, Moustafa and Alrumman, 2015).

### 3.36 *Rumex nervosus* Vahl

*Rumex nervosus* Vahl is a multi-branched shrub that can grow up to 6 feet. It is a member of the Polygonaceae family and is indigenous to North-East Africa and the Arabian Peninsula. It is known as ‘alathrub’ in Saudi Arabia, and it is most typically found in the southern portions of the country. The leaves of this plant are traditionally used to cure asthma in the area where it grows (Ali et al., 2017). Additionally, it is used in traditional practices for the treatment of headaches, eyesight impairments, arthritis, eczema, hemorrhoids, and other inflammatory disorders, among others. Different pharmacological research revealed that it possesses anti-microbial, anti-inflammatory, anti-diarrheal, anti-diabetic, and anti-oxidant properties, among other characteristics. Many classes of phytochemicals such as flavonoids, steroids and tannins was isolated from this plant. Medicinally significant chemicals such as catechin, luteolin, quercetin, and apigenin were discovered among the isolated compounds (Nigussie, 2020, Gemechu et al., 2021).

### 3.37 Solanum *surattense* Burm.f

The *Solanum surattense* plant is a low-growing shrub that may reach a maximum height of 0.4 m. It comes originally from the Southeast Asian region. It belongs to the family of plants known as the Solanaceae and may be found in India, Pakistan, and Saudi Arabia the most of the time. In Saudi Arabia, it is referred to as ‘bankum’, and it may be found in abundance in the Jizan Province, among other places. As part of traditional medicine, the whole herb of this plant is used in the treatment of asthma symptoms (Al-Shanwani 1996). According to other traditional methods, it was used as a treatment for a variety of respiratory illnesses, asthma, fever, rheumatism, chest congestion, and so on. Studies on its pharmacological properties have shown that it possesses excellent activities against malarial, fungal, bacterial infections. It is proven as very active against cancers, asthma, inflammation, ulcer, and worm infestations as well. The phytochemicals from this plant had showed excellent anti-oxidant, diuretic and wound healing properties. Secondary metabolites such as alkaloids, flavonoids, phenolic acids, triterpinoids, and fatty acids are found in abundance in this plant (Yousaf et al., 2009, Muruhan et al., 2013).

### 3.38 Solanum *coagulans* Forssk

*Solanum coagulans* is a perennial shrub with a woody base that may reach up to a height of 10 cm. This plant is a member of the Solanaceae family and is endemic to the northwest African region. Ethiopia, Uganda, Kenya, Yemen, and Saudi Arabia are just a few of the countries where it may be found. In Saudi Arabia, it is most widespread in the southwest regions, such as Wadi El-Dawaser, and it is referred to as ‘qitab’ by the locals. According to traditional treatment, cough is treated using the root and seeds of this plant (Koh et al., 2009). Other traditional practices use it to treat edema, rheumatism, arthritis, and toothache, among other ailments. Except for the detection of anti-oxidant and anti-microbial capabilities in this plant, there hasn't been much in the way of pharmacological research done on it yet. Numerous anti-oxidant compounds, including phenolic and flavonoids, have been discovered via phytochemical investigations. According to the results of phytochemical isolation, the presence of phenolic glycosides, quercetin, and radulignan was discovered (Zhang, 2015, Xu-Jie et al., 2016).

### 3.39 *Thymus vulgaris* L

*Thymus vulgaris* L. is a fragrant dwarf shrub indigenous to southern Europe. Because of its adaptation to dry and hot climatic conditions, it is widely used in many countries which belongs to the family Lamiaceae. In Saudi Arabia, this plant is exclusively found in gardens and is only observed on rare occasions. It is known as ‘za'atar’ in the local language, and it is used in traditional medicine to treat whooping cough, colds, and bronchitis by making a decoction of the leaves (Leung 1980). Additionally, it is used as a disinfectant, an anti-fungal, improves liver function, and treats urinary infections in other traditional treatments. According to pharmacological studies conducted so far, this plant has exhibited significant activities against inflammation, bacterial infections, against insecticides, virus infection among other things. It contains a number of medicinally significant phytochemicals, including thymol, Carvacrol, Eugenol, Linalool, Apigenin, and Rosmarinic Acid, among others (Prasanth Reddy et al., 2014, Hosseinzadeh et al., 2015).

### 3.40 Trianthema *portulacastrum* L

The annual *Trianthema portulacastrum* L. plant may grow either prostrate or weakly upright, and it can reach a height of up to 50 cm. It is a member of the family Aizoaceae and may be found naturally occurring in both Africa and South America*.* It may be found in large numbers in Saudi Arabia, where it is considered an invasive plant. It is known as “laani” in the area, and in the past, local therapists made use of both the root and the leaves of this plant to treat asthma in patients (Qari et al., 2021). Aside from analgesic properties, it is also used to treat bronchitis, anemia, inflammation, edema, rheumatism, as a cathartic, in the treatment of ulcers, in the treatment of jaundice and ascites. A number of scientific research have shown that this plant possesses antibacterial, anti-fungal, analgesic, hypolipidemic, hepatoprotective, anti-cancer and anti-oxidant properties. According to the results of the phytochemical investigation, it contains C-methylflavone, several water-soluble vitamins such as vitamin C and B, minerals such as copper, iron, calcium, fatty acids, and Beta-cyanin (Shivhare et al., 2012).

### 3.41 Trichodesma africanum L

The family Boraginaceae includes the multi-branched annual to short-lived perennial plant *Trichodesma africanum* L. This kind of plant is mostly found in the western tropical regions of Africa and may reach a height of 1 m. This plant may be found growing all throughout Pakistan, Iran, Egypt, and the African continent. This plant is known as ‘hamham’ in Saudi Arabia, where the entire herb is used as a traditional medication to alleviate cough (El-Ghazali et al., 2010). Other traditional uses include treating the common cold, chest congestion, ulcers, constipation, and respiratory tract infections with diuretics. It contains anti-inflammatory, antibacterial, and anti-oxidant properties, according to pharmacological investigations. Alkaloids, flavonoids, tannins, sterols, triterpenes, and anthraquinones are only a few of the pharmacologically active secondary metabolites found in it. Caryophyllene oxide, γ-eiudesmol, α-muurolene, elemol, and carvone are all present in the essential oil of this plant (El-Moaty, 2009, Abdallah Emad and El-Ghazali Gamal, 2013, Jaradat et al., 2016).

## 4 Conclusion

**Fig. 2:**
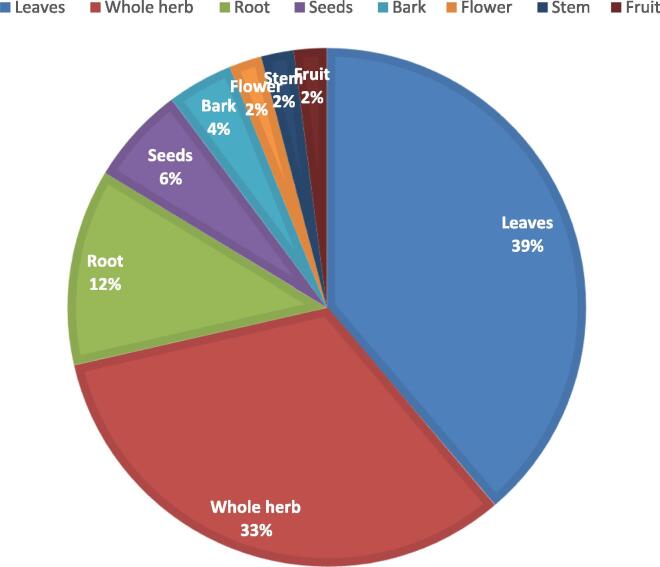
Plant parts used for respiratory diseases.

**Fig. 3:**
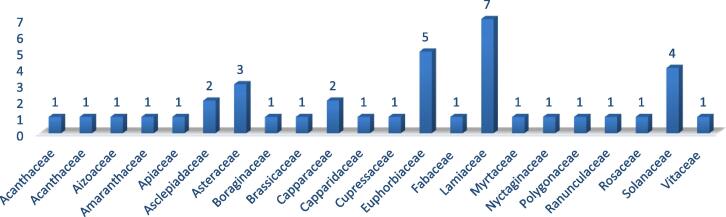
Family diversity among plants used for respiratory diseases.

It is not possible to rule out the possibility that the use of herbal treatments may play a significant role in the healthcare business in the years to come. It has widespread use and has shown efficacy in a broad range of settings. It should come as no surprise that diverse regions of the globe have developed their own unique sets of customs and rituals. These techniques are dependent on the materials and plants that are available in the location where they are carried out. The vast majority of the time, neither the documentation nor the scientific data can back them up. There is a category of ailments known as respiratory tract diseases. The severity of these diseases may vary from mild symptoms to life-threatening conditions. In addition, the factors that led to this result are complex and multi-faceted in character. In general, it is suggested to treat the symptoms of such diseases as well as the underlying cause of the condition. Herbal treatments have shown great efficacy in both of these circumstances; in fact, several of these herbal medicines are already in use in clinical settings and are available for purchase in retail outlets. During the process of carrying out the current study, it was essential to collect data on those plants that have comprehensive information readily available in the relevant published sources. According to the research, there are 41 distinct types of plants that have been historically used to treat a range of respiratory illnesses in Saudi Arabia. These plants are employed in traditional medicine. Every component of plants is harvested for use; however, leaves account for over 40 % of all plant components consumed, followed by complete herb formation (33 %) (Fig. 2). The Lamiaceae family is responsible for the discovery of the largest proportion (7 %) of new plant species in this investigation, with the Euphorbiaceae family coming in at a distant second (5 %) (Fig. 3). According to the research, the most common use of plants is in the treatment of asthma (21), followed by cough management (18). It was observed that there are six plants that are used in the treatment of bronchitis, and it was found that there are four plants that are used in the treatment of the common cold. It was found that the remaining herbs have the ability to cure a variety of respiratory conditions as well as the symptoms associated with those conditions. Take into consideration the fact that the great majority of plants included in this investigation have not been subjected to any kind of scientific research on respiratory problems, either in the lab or in clinical trials. This is important to keep in mind. In the specific domain of traditional plants, it demonstrates that there is a significant gap between ethnopharmacological research and current knowledge. It is also important to emphasize the possibility of conducting a scientific research on these plants for respiratory problems in order to have a better understanding of both the beneficial and detrimental effects of these plants have. The availability of scientific evidence further enhances the possibility of commercializing these plant products.

## Declaration of Competing Interest

The authors declare that they have no known competing financial interests or personal relationships that could have appeared to influence the work reported in this paper.
